# Clinical and molecular characterization of 1q43q44 deletion and corpus callosum malformations: 2 new cases and literature review

**DOI:** 10.1186/s13039-022-00620-2

**Published:** 2022-10-03

**Authors:** Bochra Khadija, Khouloud Rjiba, Sarra Dimassi, Wafa Dahleb, Molka Kammoun, Hanen Hannechi, Najoua Miladi, Neziha Gouider-khouja, Ali Saad, Soumaya Mougou-Zerelli

**Affiliations:** 1 Laboratory of Human Cytogenetics, Department of Human Cytogenetics, Molecular Genetics and Biology of Reproduction, Farhat Hached University Hospital, Sousse, Tunisia; 2 Higher Institute of Biotechnology, Monastir University, Monastir, Tunisia; 3 Common Service Units for Research in Genetics, Faculty of Medicine of Sousse, University of Sousse, Sousse, Tunisia; 4 Medical Maghreb, El Manar 3, 2092 Tunis, Tunisia; 5 University of Tunis El Manar, 2092 El Manar 1, Tunis, Tunisia; 6 Head of Department at the National Institute of Neurology Tunis Head of RU On Movement Disorders, Tunis, Tunisia

**Keywords:** Corpus callosum agenesis, Chromosomic abnormalities, 1q43 microdeletion syndrome, Array CGH

## Abstract

**Background:**

Corpus callosum malformations (CCM) represent one of the most common congenital cerebral malformations with a prevalence of around one for 4000 births. There have been at least 230 reports in the literature concerning 1q43q44 deletions of varying sizes discovered using chromosomal microarrays. This disorder is distinguished by global developmental delay, seizures, hypotonia, corpus callosum defects, and significant craniofacial dysmorphism. In this study, we present a molecular cytogenetic analysis of 2 Tunisian patients with corpus callosum malformations. Patient 1 was a boy of 3 years old who presented psychomotor retardation, microcephaly, behavioral problems, interventricular septal defect, moderate pulmonary stenosis, hypospadias, and total CCA associated with delayed encephalic myelination. Patient 2 was a boy of 9 months. He presented a facial dysmorphia, a psychomotor retardation, an axial hypotonia, a quadri pyramidal syndrome, a micropenis, and HCC associated with decreased volume of the periventricular white matter. Both the array comparative genomic hybridization and fluorescence in situ hybridization techniques were used.

**Results:**

Array CGH analysis reveals that patient 1 had the greater deletion size (11,7 Mb) at 1q43. The same region harbors a 2,7 Mb deletion in patient 2. Here, we notice that the larger the deletion, the more genes are likely to be involved, and the more severe the phenotype is likely to be. In both patients, the commonly deleted region includes six genes: *PLD5, AKT3, ZNF238, HNRNPU, SDCCAG8* and *CEP170*. Based on the role of the *ZNF238* gene in neuronal proliferation, migration, and cortex development, we hypothesized that the common deletion of *ZNF238* in both patients seems to be the most responsible for corpus callosum malformations. Its absence may directly cause CCM. In addition, due to their high expression in the brain, *PLD5* and *FMN2* could modulate in the CCM phenotype.

**Conclusion:**

Our findings support and improve the complex genotype–phenotype correlations previously reported in the 1qter microdeletion syndrome and define more precisely the neurodevelopmental phenotypes associated with genetic alterations of several genes related to this pathology.

## Background

Corpus callosum malformations (CCM) represent one of the most common congenital cerebral malformations with a prevalence of around one for 4000 births.

Any defect during the critical period of corpus callosum formation leads to an abnormal development resulting in malformations ranging from complete agenesis to structural defects (hypoplasia and dysplasia). Furthermore, the severity of the nature of the CC defect depends essentially on the time of onset of the etiological factor. These malformations can occur isolated or in association with chromosomal, syndromic, or monogenic disorders; or, rarely, secondary to infectious, ischemic, or teratogenic causes.

1qter rearrangements lead to recognizable and large phenotype due to the size of the segment and the number of genes involved. The clinical features include severe mental retardation, profound growth retardation, microcephaly, and an abnormal corpus callosum. The extreme clinical variability has been ascribed to the contiguous genes located in the deleted region, but specific genotype/phenotype correlations remained uncertain until now.

However, the recent use of microarray analysis has allowed the detection of various submicroscopic alterations as well as the fine mapping of deletion sizes in individuals with distinct or variable clinical features. Nevertheless, identification of the specific gene(s) responsible for the various features has remained elusive because of the conflicting reports suggesting, that the etiology might be more complex or additional high-resolution array data in conjunction with accurate genotype–phenotype correlations in more cases are needed.

Chromosome 1q44 deletion was first described in 1976 by Mankinen et al. [1]. Up to now, there are at least 232 cases of deletion in 1q43q44 or 1q44 have been reported in the literature with a variability of sizes identified by chromosome microarray [2].

In this paper, we describe two patients with 1q microdeletions at 1q43q44 and 1q42q44, characterized by array comparative genomic hybridization (aCGH), and attempt to establish genotype–phenotype correlations, aiming to bring further insight into the gene’s implication in this region in brain abnormalities.

## Results

**Fig. 1:**
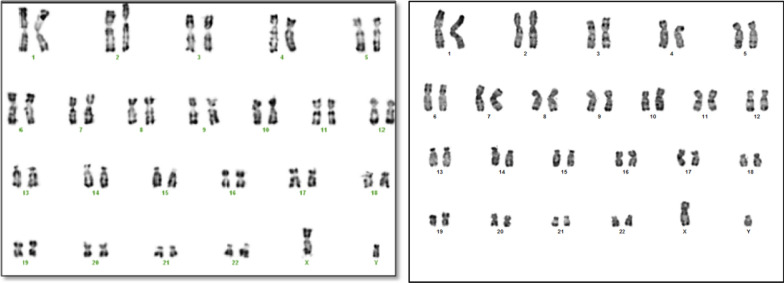
The karyotype on the left **a** is for the patient 1 who has asymmetry on 1q and the karyotype on the right **b** is for patient 2

**Fig. 2:**
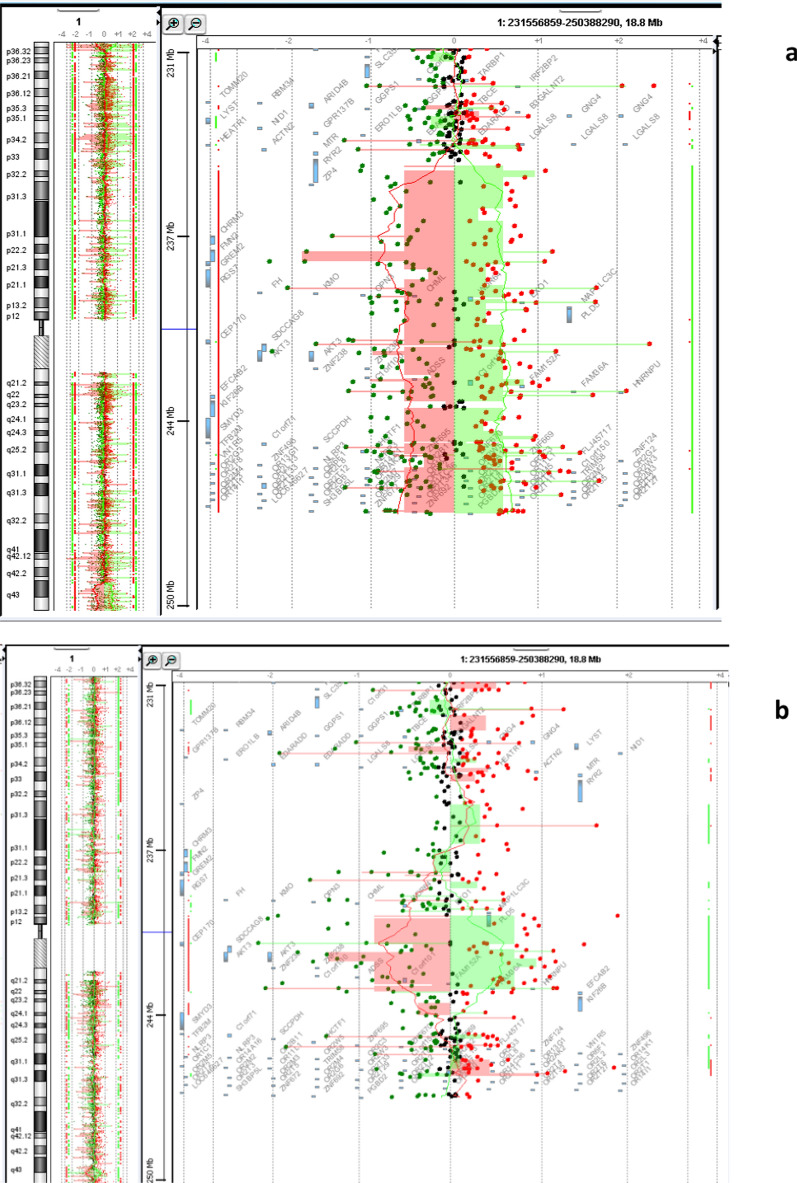
44,0000 Agilent Technologies oligonucleotides array profiles of both patients showing: **a** deletion of at least 11,7 Mb in patient 1. **b** deletion of at least 2,7 Mb in patient 2

**Fig. 3:**
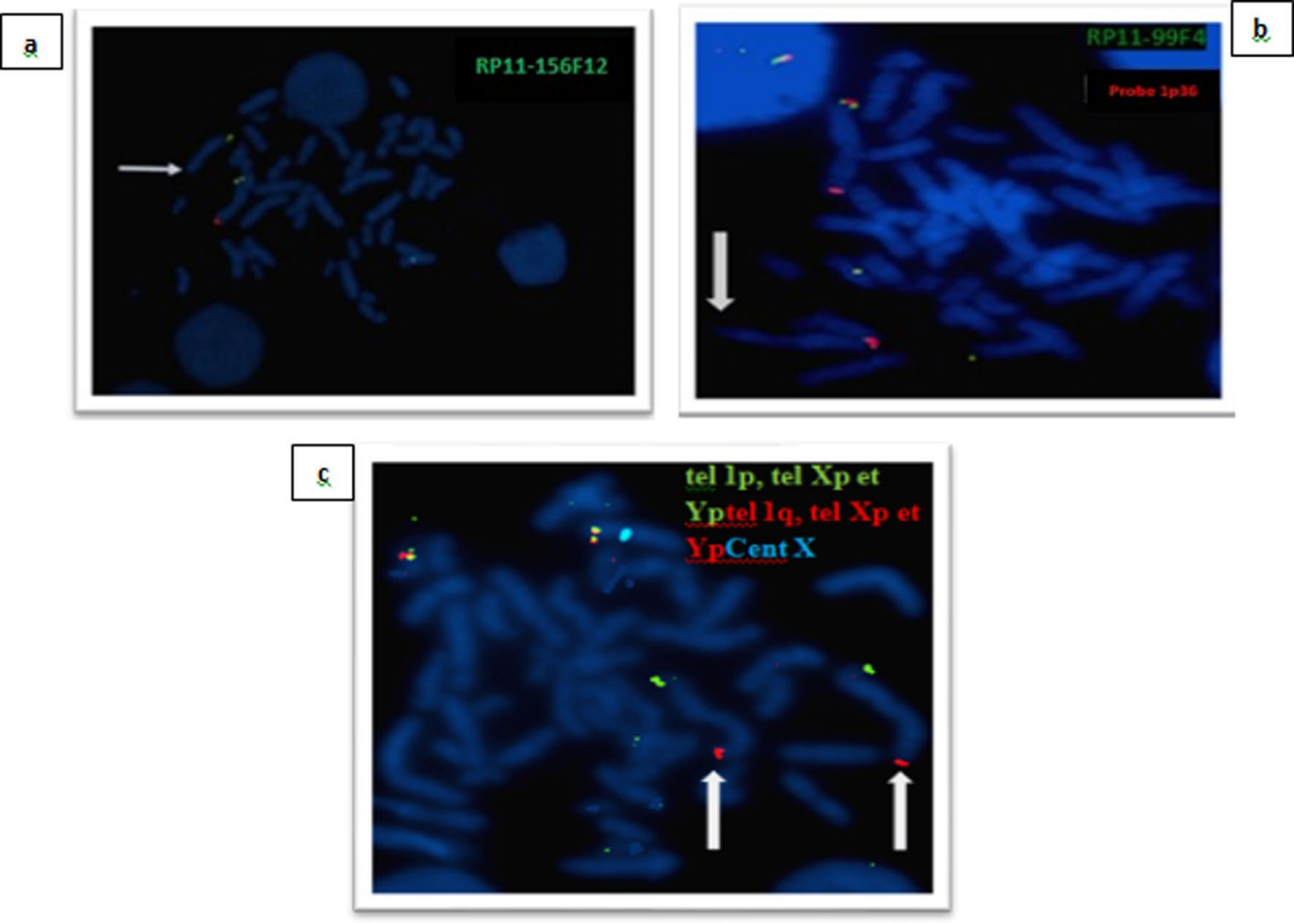
FISH analysis with commercial probes (**a**: Bluefish probe of telomere 1q: corresponding to the *CEP170* gene and a control probe 1p36 in patient 1, **b**: green fluorescence probes corresponding to the *PLD5* gene and a 1p36 red fluorescence control probe, **c**: confirmation of the interstitial deletion by performing FISH on the two q telomeres of the two homologous chromosomes 1 in patient 2). The white arrow shows the terminal deletion in patient 1 (**a**) and the interstitial deletion in patient 2 (**b**, **c**)

For the first patient, the conventional karyotype revealed a male karyotype with an asymmetry on 1q which is represented by a minor difference in the size of the two 1 chromosomes in the long arm q (Fig. 1a). Array CGH characterized a terminal deletion of the long arm of chromosome 1 encompassing at least 11,7 Mb extending from nucleotide 235,500,506 to 247,179,291 according to the Human reference genome hg18, 46,XY.arr[hg18] 1q42.3(235,500,506_247,179,291) × 1 dn (Fig. 2a). This deletion was well confirmed by FISH with a Bluefish probe of telomere 1q: corresponding to the *CEP170* gene and a control probe 1p36 (Fig. 3a).

Then, karyotypes of the parents, as well as FISH using the same probes, didn’t reveal any chromosomal anomalies within the limits of resolution. These results indicate the de novo character of this anomaly.

The chromosomal analysis of the second patient indicates a normal male karyotype 46, XY in all metaphases (Fig. 1b). Array CGH analysis revealed an interstitial deletion of the long arm of chromosome 1 encompassing at least 2.7 Mb ranging from nucleotide 240,354,387 to 243,090,301 according to the Human reference genome hg18, 46,XY.arr[hg18]1q43(240,470,722_243,198,799) × 1 dn (Fig. 2b).

In addition, the FISH assay confirmed the chromosomal rearrangement with the green fluorescence probes corresponding to the *PLD5* gene and a 1p36 red fluorescence control probe. And the interstitial character of the deletion is identified by performing FISH on the two q telomeres of the two homologous chromosomes 1 (Fig. 3b). Our clinical and genetic results are resumed in Table 1.

**Table 1:** Phenotypic features of the two patients with deletion in 1q44 described in this study and those published earlier

References	Boland et al. 2007 [3]	Hill et al. 2007 [4]	Caliebe et al. 2010 [5]	Zaki et al. 2012 [6]	Nagmani et al. 2012 [7]	Thierry et al. 2012 [8]	Ballif et al. 2012 [9]	Parlman et al. 2013 [10]	Gupta et al. 2013 [11]	Van et bon et al. 2008 [12]	Gai et al.,2015 [13]	Hemming et al. 2016 [14]	Depienne et al. 2017 [15]	Mohamed et al. 2018 [16]	Córdova-Fletes et al. 2019 [17]	Lloveras et al. 2019 [18]	Present study	
Number of Patients	6	7	4	1	6	11	22	1	1	3	1	1	17	1	1	1	1	2
Gender	3F/4 M	5F/2 M	3F/1 M	M	4F/2 M	8F/3 M	9 M/ 13F	F	F	M	M	M	10F/7 M	F	M	M	M	M
Age	2 -5 years	6–18 years	1- 2 years	2 years	2–17 years		1–14 years	2 years	3 years	14 years	5 and ½ years old	14 years	1–22 years	3 years	3years2M	9 years	3 years old	9 months
FD	+	+	+	RF, PM, MIC, TUL, LSE, SYN	+ in 3/6	+	+	RF, TL, EP SFH, PM, SN, MIC, USP, T, LSE, WST	RF, HYP, SN	MIC, SN, TL, EP, USP, S, LSE, WST	RF, SN, USPF, WST, LSE, T	MIC, EP, USPF	+	RF, SFH, PM, MIC, SN, TL, USPF, LSE, WST	RF, SFH, PM, MIC, SN, T, USPF, WST, LSE,	-	RF, SFH, PM, SN, TL, EP, USPF, S, T, LSE, WST	RF, SFH, PM, HYP, MIC, SN, TL, EP, LSE, WST
Microcephaly	4/6	6/7	¾	+	5/6	1,2	7/22	-	+	+	+	+	+	+	+	+	+	+
DD/ID	+	6/7		+	6/6	+	–	–	+	+	+	–	+	+	+	+	+	-
GR	–	3/7		–	–	–	–	+	–	–	–	–	–	–	+	+	+	+
PD	+	–		+	–	+	–	+	+	+	+	–	+	+	+	+	+	+
LR	–	–	4/4	+	–	5/11	–	–	–	+	+	–	+	+	–	+	+	-
BP	–	–		-	2/6	4/11	–	–	–	+	–	–	–	+	–	+	+	-
Epilepsy	+	6/7	4/4	+	2/6	11/11	10/22	–	+	+		+	+	+	–	+	–	+
Axial hypotonia	–	–	4/4	+	1/6	4/11	+	+	–	+	+	+	–	–	+	–	–	+
FD	–	–	–	–	–	–	–	+	–	+	+	+	–	–	–	–	–	–
GA	–	–	1/4, Hypos	Hypog, AP	–	2/11 Crypto	–	–	–	–	–	–	–	–	Bilateral crypto	–	hypos	Micropenis TE
CM	–	–	2/4 VSD, ASD	–	3/6 ASD, TOF, VSD	1/11	–	–	–	–	–	–	–	–	VSD, ASD	–	ISD, PS	–
OM	–	–	–	Clino	–	–	–	SHF	Polydac	Scoliosis	–	Scoliosis	–		Campto, GV, TV	–	Polydac	Polydac
Brain IR	ACC: 5/6	ACC: 6/7	ACC in 4/4	ACC	ACC in 4/6	ACC in 1/11	ACC in 7/22	HCC Pach, Vent	HCC	HCC	Nle CC	P ACC	ACC in 15/17	HCC	ACC	HCC	T CCA BA,DEM	HCC DVPWM
Deleted region	1q42q44	1q43q44	1q43q44	1q43q44	1q43q44	1q44	1q43q44	1q44	1q44	1q43q44	1q43q44	1q43q44	1q43q44	1q43q44	1q43q44	1q43q44	1q43q44	1q42q43
Deletion size (Mb)	3,5	2	0,44	10,4	1,4	0,63	2	1,47	1,86	3,8	0,36	8	1,36	6,5	9,5	2,3	11,7	2,7
GP: Start hg 18/19 (pb)	243,100,000	243,000,000	244,968,377	238,681,384	242,987,737	244,900,000	243,433,377	242,191,892	244,744,522	-	243,651,534	241,183,190	243,100,00	242,664,760	239,721,730	243,011,722	235,500,506	240,470,722
GP: End hg 18/19 (pb)	245,715,000	245,000,000	245,394,377	249,190,989	244,331,570	245,100,000	245,433,377	243,660,791	246,608,189	-	24,4014,380	249,202,755	244,500,000	249,206,918	249,218,792	245,384,463	247,179,291	243,198,799
Involved genes	*AKT3, CEP170, ZNF238*	*CEP170, SDCCAG, AKT3*	*FAM36A HNRNPU, EFCAB2, KIF2613*	*PLD5, FMN2, RGS7, AKT3, ZNF238, HNRNPU, SMYD3*	*CEP170, ZNF238, SDCCAG8*	*HNRNPU, FAM36A,*	*AKT3, ZNF238, FAM36A, CIORF199, HNRNPU*	*PLD5 CEP170*	*HNRNPU SMYD3*	*PLD5, AKT3, ZNF238 CEP170*	*AKT3*	*PLD5, CEP170, SDCCAG8, AKT3, KMO ZNF238, HNPNPU*	*AKT3, HNRNPU, ZNF238*	*CEP170, AKT3, ZNF238, HNRNPU*	*AKT3, ZNF238, HNRNPU*	*AKT3, ZNF238, SDCCAG8, HNRNPU*	*AKT3, PLD5, ZNF238, SDCCAG8 FMN2, RGS7, SMYD3,KMO,HNRNPU, CEP170*	*AKT3, PLD5, SDCCAG8, ZNF238, CEP170 HNRNPU,*
Inheritance	–	*–*	De novo	De novo		De novo	De novo	De novo	–	De novo	–	De novo		–	–	–	De novo	De novo

ACC: agenesis of corpus callosum, ASD: Atrial Septal Defect, BP: Behavioral Problems, BA: Brain Atrophy, CC: Corpus Callosum, CM: Cardiac Malformations, Clino: Clinodactyly, Campto: Camptodactyly, Crypto: Cryptorchidy, DD: Development Delay, DEM: Delayed Encephalopathic Myelination, DVPWM: Decreased Volume of the Periventricular White Matter, EP:Epicanthus, F: female, FD: Facial Dysmorphia, FD: Feeding Difficulties, GA: Genomic Positions, GA: Genital Anomalies, GV: Genu Valgum, Hypog: Hypogenitalism, Hypos: Hypospadias, HCC: Hypoplasia of corpus callosum, HYP: Hypertelorism, ID: intellectual deficiency, IR: Imaging Results, LR: Language Retardation, MIC:Micrognathia, M: male, OM: Other Malformations, PM: Protrusion of the metopic, RF: Round Face, TV: Talipes Valgus, TOF: Tetralogy *of* Fallot, TE: Testicular Ectopia, T ACC: Total Agenesis of the corpus callosum, P ACC: Partial Agenesis of the Corpus Callosum, Pach: Pachygyria, Vent: Ventriculomegaly, SFH: Short forehead, SN:Short nose with a broad root, THL: Thin upper lip, T:Telecanthus, UPSPF: Up slanted palpebral fissure VSD: *ventricular septal defect*, S:Strabismus, LSE: Low-set ears, WST: widely spaced teeth

## Discussion

Identifying candidate genes and subsequently, correlating their biological function to specific clinical phenotypes remains an enduring challenge in medical genetics. During the past decades, the detection of 1q deletion and its genotype–phenotype correlation had been limited due to low-resolution techniques (G-band karyotype, FISH, satellite marker analyses…).

However, the recent use of array CGH allows us to specifically pinpoint breakpoints and more accurately define a deletion and its contents [18]**.**

The 1q43q44 deletion is a rare chromosomal disorder that frequently goes undiagnosed. However, it results in a distinct clinical disorder phenotype characterized by moderate to severe intellectual disability, psychomotor retardation, corpus callosum agenesis or hypoplasia, epilepsy, and a distinctive craniofacial dysmorphia consisting of microcephaly, a round face, a thin upper lip with a prominent cupid's bow, thin, downturned corners of the mouth, smooth philtrum, micrognathia, retrognathia, short Hands and feet abnormalities are also possible. Abnormalities of the hands and feet can also occur. Growth retardation with short stature is a common feature. Furthermore, congenital malformations such as cardiac, skeletal, and urogenital defects have been recorded (in male patients) [2, 19]**.**

Our study is based on the analysis of two patients with corpus callosum malformations, which are characterized by genetic and phenotypic heterogeneity. Two terminal 1q deletions with different breakpoints and different size deletions were detected.

The first one, a 3-year-old child followed for psychomotor retardation, total agenesis of the corpus callosum, microcephaly, an obvious facial dysmorphia, polydactyly, hypospadias, an interventricular septal defect associated with moderate pulmonary stenosis and behavioral problems such as manual and verbal stereotypy. Genetic explorations in this patient had shown deletion of the estimated size of 11.7 Mb in 1q42q44. The first patient's deletion contains several genes, including ten OMIM genes with high brain expression: *RGS7, FMN2, PLD5, SDCCAG8, AKT3, ZNF238, CEP170, HNRNPU, and SMYD3.*

The second patient was a 9-month-old infant with CC hypoplasia, psychomotor delay, axial hypotonia, polydactyly, testicular ectopia, micropenis, seizures, and facial dysmorphia. This deletion encompassing 2,7 Mb affects six OMIM genes: *PLD5, SDCCAG8, AKT3, ZNF238, HNRNPU, and CEP170*. These genes are shared between the two patients. The *ZNF238* and the *AKT3* genes represent the two genes included in the CCM minimal critical region and the deleted region in both patients.

**Fig. 4:**
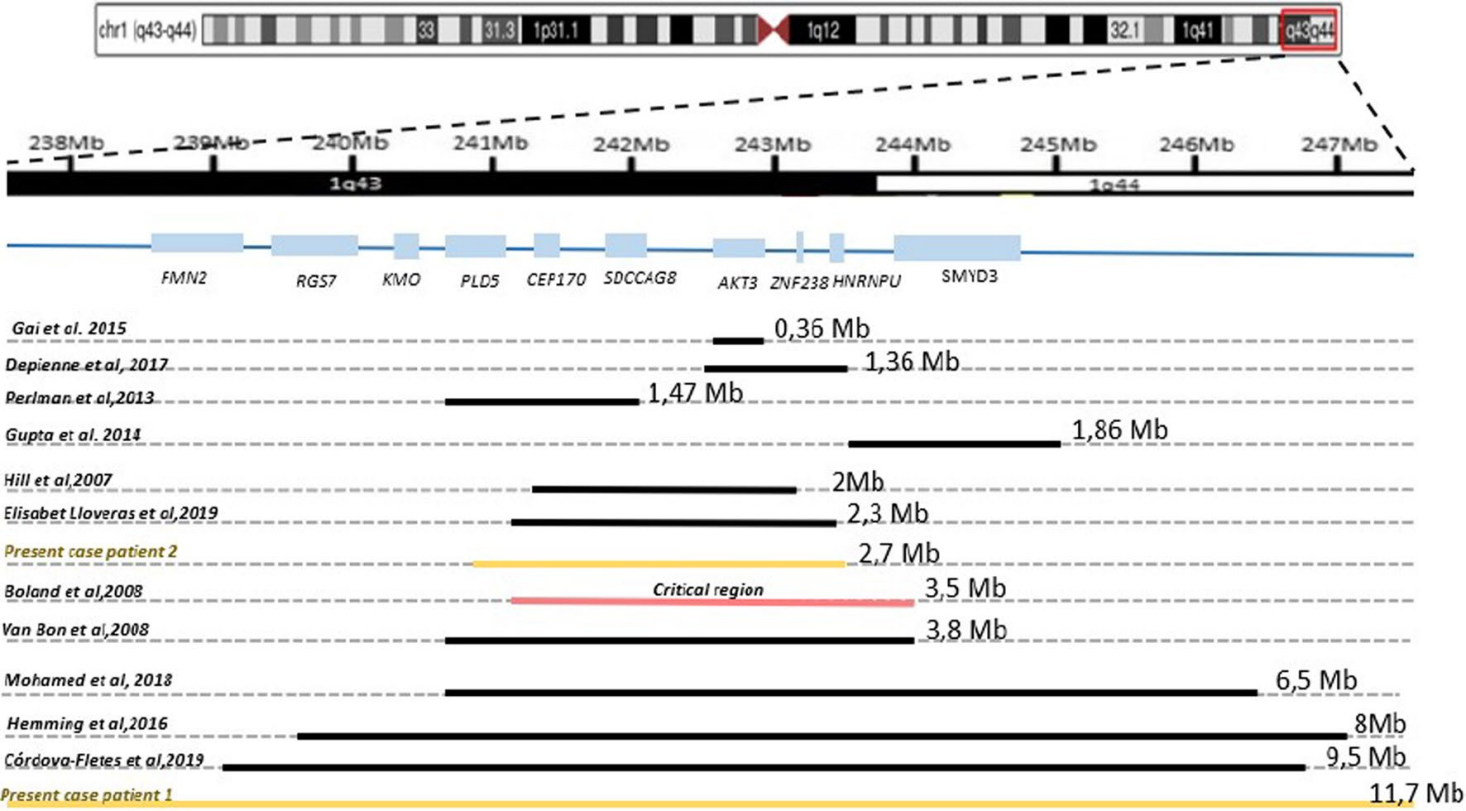
Comparison of the size of the 1q deleted region, critical region, and involved genes in our patients and other studies. A schematic representation of the gene mapping at the 1q43q44 region is shown below the human chromosome pictogram

So far, the genotype–phenotype correlation of the *AKT3* gene was not obvious. Some studies affirmed its involvement in CCM while others didn’t. Indeed, this gene was considered as a candidate gene for CCA based on a critical region of 3,5 Mb (as mentioned in Fig. 4) previously described in a cohort of 6 patients [3]**.**

The *AKT3* gene (OMIM 611,223; v-akt murine thymoma viral oncogene homolog 3 gene) encodes a serine/threonine-kinase and was found to be involved in brain development in mice [20]**.** Members of the AKT protein family, such as *AKT3*, are implicated in numerous biological processes, including adipocyte and muscle differentiation, glycogen synthesis, glucose uptake, apoptosis, and cellular proliferation (OMIM 611,223) [18]**.** However, Akt3-/- KO mouse models exhibit reduced brain size and hypoplasia of the corpus callosum.

However a few years later, two-terminal 1q chromosomal deletions with CCA cases of 360 Kb and 1,47 Mb respectively that do not contain the *AKT3* gene rule out its involvement in this phenotype [3, 10]**.** Interestingly, microcephaly has been reported in nearly the half of 1q43q44 deletion patients and suggesting that *AKT3* haploinsufficiency is the main cause of microcephaly in this syndrome [7–9]**.** These findings are consistent with this study in both patients. This emphasizes the association *AKT3* -microcephaly in the 1q44microdeletional syndrome rather than its links with CCA.

1q43q44 submicroscopic deletions have been associated to brain disorders and particularly CCM. To the best of our knowledge, all of them harbor the *ZNF238* gene. This gene encodes a Zinc-finger type-C2H2 protein acting as a transcriptional and chromatin regulator highly expressed in the developing and adult brain. Additional studies of the spatiotemporal expression of *ZNF238* (OMIM-608433: called also *ZBTB18)* suggest that it plays a crucial role in neuronal proliferation, migration, and development in the cortex [21].

Moreover*, Znf238*-mutant brains maintained neuronal precursor pools but had decreased neuronal and increased glial differentiation. Indeed, *ZNF238* seems to promote neuronal differentiation and brain growth by repressing multiple pro neurogenic genes such as *Ngn2* and *Neurod1*. Thus, it plays a significant role in the promotion of ordered neurogenesis leading to proper layer formation and cortical growth. The mutant mice's phenotype was similar to that of patients with chromosome 1q43-q44 deletion syndrome [22]**.** This gene was particularly found to be highly relevant to CCA [23, 24]**.**

Sismani C, et al. described a patient with a 8 Mb terminal deletion in 1q43q44 [25]. This deletion contains the genes we studied, particularly the *ZNF238* gene. Furthermore, the patient showed the main clinical features of the 1q43q44 microdeletional syndrome but no corpus callosum defects. This can be explained by additional mechanisms such as incomplete penetrance, variable expressivity, or multigenic factors may also influence phenotypic expression. However, it was suggested that co-deletion of multiple genes in contiguous gene syndromes may have additive effects [15]**.**

In a report on 17 patients with 1q43q44 microdeletions, the authors focused on three genes: *AKT3, HNRNPU,* and *ZNF238*. These three genes are involved in the most clinical manifestation of 1q43q44 deletion and are significantly expressed in the brain and play a significant role in the most severe clinical manifestation of 1q43q44 deletion. They discovered a 1.36 Mb critical region (as showed in Fig. 4) that contains the selected genes. Furthermore, four of the 17 patients studied had *ZNF238* mutations and seven had *HNRNPU* mutations [15]**.**

*HNRNPU* (OMIM 602,869) encodes a highly conserved protein that binds RNAs and mediates their metabolism and transport. It regulates embryonic brain development. Several studies argue that *HNRNPU* is the main gene responsible for seizures in patients with 1q43q44 haploinsufficiency [9, 15].

Even if in some cases (Table 1), the deletion did not encompass all three genes mentioned above, the common deleted interval in both patients in the present study encloses the *CEP170* gene, a gene reported as deleted in 1q44 deletion syndrome in several studies [4, 7, 10, 15, 26]**.** *CEP170* gene is expressed extensively in the brain and encodes for a centrosomal complex protein. known for their neurogenesis function and so as hypothesized before in human brain size also explaining its additional role in microcephaly [7]**.**

The *SDCCAG8,* gene encoding a centrosome-associated protein, was also included in the commonly deleted region in both patients. It may act during interphase and mitosis in centrosome organization. Interestingly, this gene is associated with two autosomal recessive ciliopathies Bardet Biedl and Senior Loken syndromes (OMIM: 615,993 and OMIM: 613,615, respectively) which emphasis the centrosomal role of this gene.

Curiously, the heart defect noted in patient 1 could be attributed to the *SMYD3* gene haploinsufficiency. This gene highly expressed in fetal heart development is located beyond the CC critical region. It should be noted that both Smyd3 knockout and knockdown models were related to various cardiac defects [27]**.**

Furthermore, the *FMN2* gene encodes for cytoskeletal organization and establishment of cell polarity and is expressed exclusively in the central nervous system specifically in the cerebral cortex, pituitary gland, and spinal cord [28]**,** which could explain its involvement in CC development. It was otherwise associated with unexplained infertility [29] or leukemia [30]**.** We also hypothesize a correlation between alteration of this gene and psychomotor delay, and our hypothesis supports previously reported results [28]**.**

The *FMN2 and RGS7* genes are not known to be associated to corpus callosum anomalies [31]**.** *RGS7* is a regulator of G-protein signaling 7 which may play a role in synaptic vesicle exocytosis and the rapid regulation of neuronal excitability. But interestingly, *FMN2 and RGS7* deletion was discovered in Warburg–Micro Syndrome patients. The syndrome’s common features include a microcephaly, a congenital cataract, a central nervous system malformation (particularly CCA/HCC), a severe intellectual disability, a growth failure, a progressive contracture, and a facial dysmorphism.

As seen in Fig. 4, the commonly deleted region (in both patients involves *PLD5,* a phospholipase D highly expressed in the brain and involved in cytoskeletal and transcriptional regulation. *PLD5* is still a poorly understood gene, and seems to be associated to autism spectrum disorder in the first reports. Further, copy number variations including the *PLD5* gene were associated to brain disorders but described also as simply "passenger genes," since its variations were found in normal individuals in the Database of Genomic Variants [32]**.**

Even though, its deletion in both patients of this study is linked to severe symptoms. Indeed, some 1q43 deletions containing *PLD5, ZNF238, AKT3, and HNRNPU* harbor severe phenotypes [14]. In addition, *PLD5*'s position upstream of the CC defects critical region and the few studies that include it as a causal gene in CCM, and based on its function as a transcriptional factor, may indirectly contribute to CCM by modifying the activity of other genes found in distant locations.

Taken together, we can suggest that the *ZNF238* haploinsufficiency gene is the most likely pathogenic mechanism for the CCA in patients with such deletions. And so, the critical region for corpus callosum defects, containing the *ZNF238* gene and *AKT3*, is under the transcriptional control of a region containing the *PLD5* gene as seen in both patients.

To summarize in this study, the deletion in both patients includes the minimal critical region for corpus callosum defect, explaining their phenotype, which is consistent with what has been described in the literature. The absence of reciprocal translocation or pericentric inversion in the parents defined this deletion as pure and de novo. In summary, we suggest *ZNF238* as an intriguing candidate gene for contribution to the corpus callosum anomalies. *PLD5* may also be implicated in CCM due to its high expression in the brain.

## Conclusion

In conclusion, CCM presents a multigenic pathology with a spectrum of phenotypic features, from very subtle and mild to a wide range of severe aberrations. Normal development of the brain depends on highly regulated spatiotemporal gene expression, and a normal corpus callosum development seems to rely on several genes and elements in the 1q44 region.

Array CGH allowed us to better identify breakpoints and genes likely to be involved in this deletional syndrome. To understand the genotype–phenotype correlation, the reports of patients with deletion of different genes and regions provide important information.

As a result, our work confirms the implication of *ZNF238* in CCM and highlights new candidate genes such as *PLD5, and FMN2.* However, to further improve our understanding of the function, regulation, and interaction of these genes, also experimental and functional studies will be required in the future.

## Patients and methods

### Patients

**Fig. 5:**
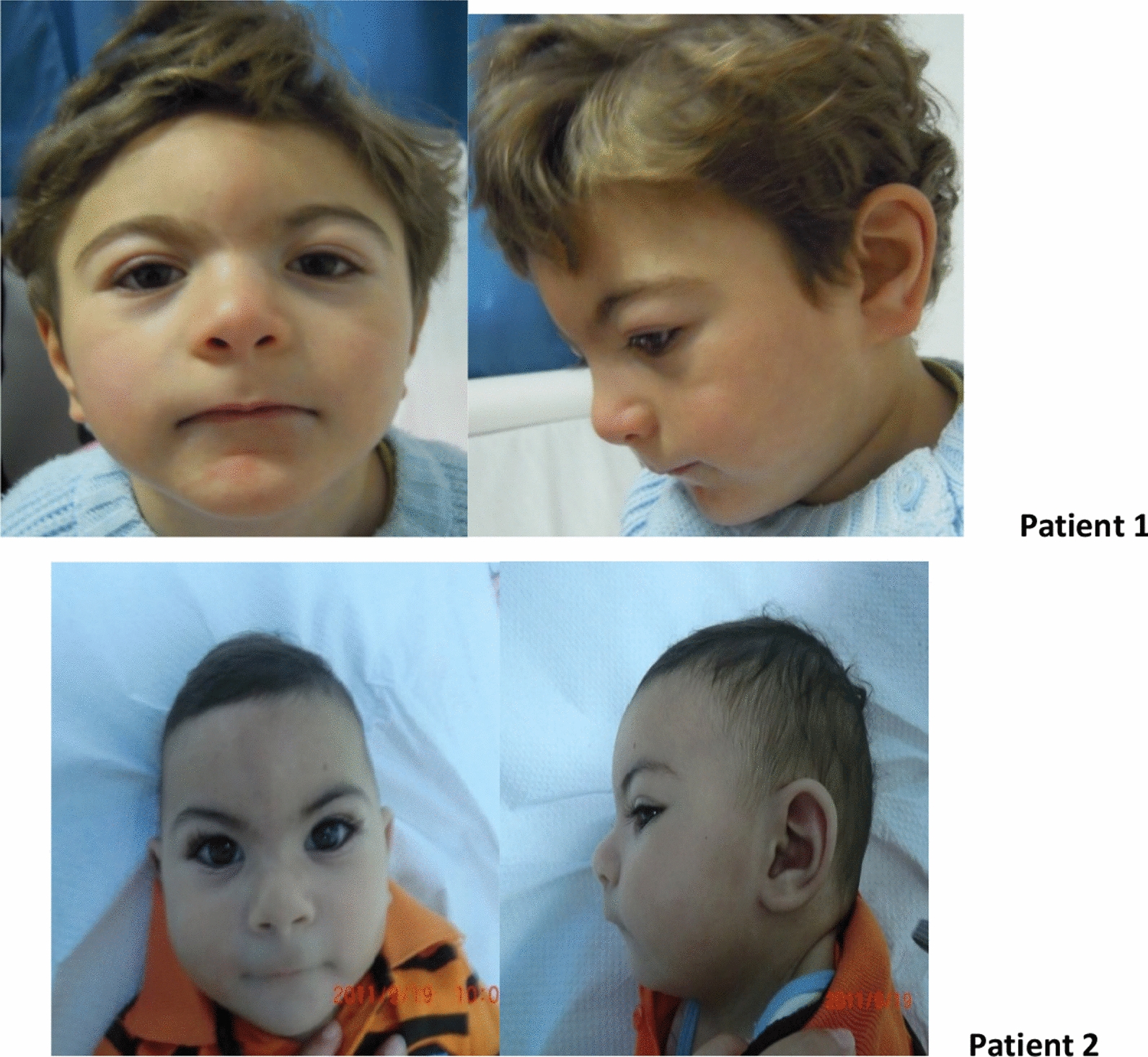
showing facial dysmorphia in both patients

The two patients were referred to the Cytogenetics and Reproductive Biology Department of the Farhat Hached Hospital in Sousse for cytogenetic analysis to explore the genetic origin of their developmental delay and/or intellectual disability, brain malformations, and facial dysmorphia. The clinical results are shown in (Table1), and photographs are presented in (Fig. 5).

## Methods

### Karyotype

Our patients have benefited from a conventional R-band karyotype at a 450-band resolution of a first intention. According to a standard protocol, metaphase chromosome spreads were prepared from phytohemagglutinin-stimulated peripheral blood lymphocytes. Cell cultures were, incubated for 72 h. At least 25 mitoses were studied for each sample using Cytovision® karyotyping software version 4.0. Karyotypes were classified according to the international system of Human cytogenetic nomenclature (ISCN2020)[33].

### Fluorescent in situ hybridization (FISH)

FISH was performed on blood lymphocytes blocked on metaphases of each patient, according to the standard protocol. FISH followed manufacturer’s instructions, using probes for chromosome 1 (RP11-99F4 (1q43; *PLD5*; 1p36 for patient n°2 and RP11-156F12 (1q43; *CEP170*; 1p36 for patient n°1). Probes were applied to metaphase slides and therefore co-denaturized for 7 min at 75 °C. After overnight hybridization at 37 °C, the slides were washed for 5 min in de 2XSSC/ NP40 (Vysis, Illinois, Unites States) at 75 °C. Chromosomes were mounted with a 4,6 diamino-2-phenylindole and analyzed using an Axioskop Zeiss® fluorescent microscope.

### Array CGH

Array comparative genomic hybridization (array CGH) was performed with Agilent Human Genome array CGH Kit 44 K, for both patients. The coverage of the human genome was made with an average spatial resolution of 75,000 pair bases. A copy number variation was noted when at least three contiguous oligonucleotides presented an abnormal ratio greater than + 0.58 or lower than − 0.75. In-silico analysis of the unbalanced regions was made using the UCSC Genome Browser (https://genome.ucsc.edu/), the Database of Genomic Variants (DGV: http://dgv.tcag.ca/dgv/app/ home), and the Online Mendelian Inheritance in Man database (OMIM: https://omim.org/).

### Clinical description

#### Patient 1

A boy aged 3 years old and a half and from a non-consanguineous marriage. In his personal history, we noted intrauterine growth retardation (IUGR). He was hospitalized in the neonatal period for poly malformative syndrome. He presents a psychomotor delay with the acquisition of the sitting position at 10 months and the standing position with support at 2 years and a half. He still does not speak. The child also has behavioral problems such as manual and verbal stereotypies, self-aggression, anger, and unexplained laughs.

**Fig. 6:**
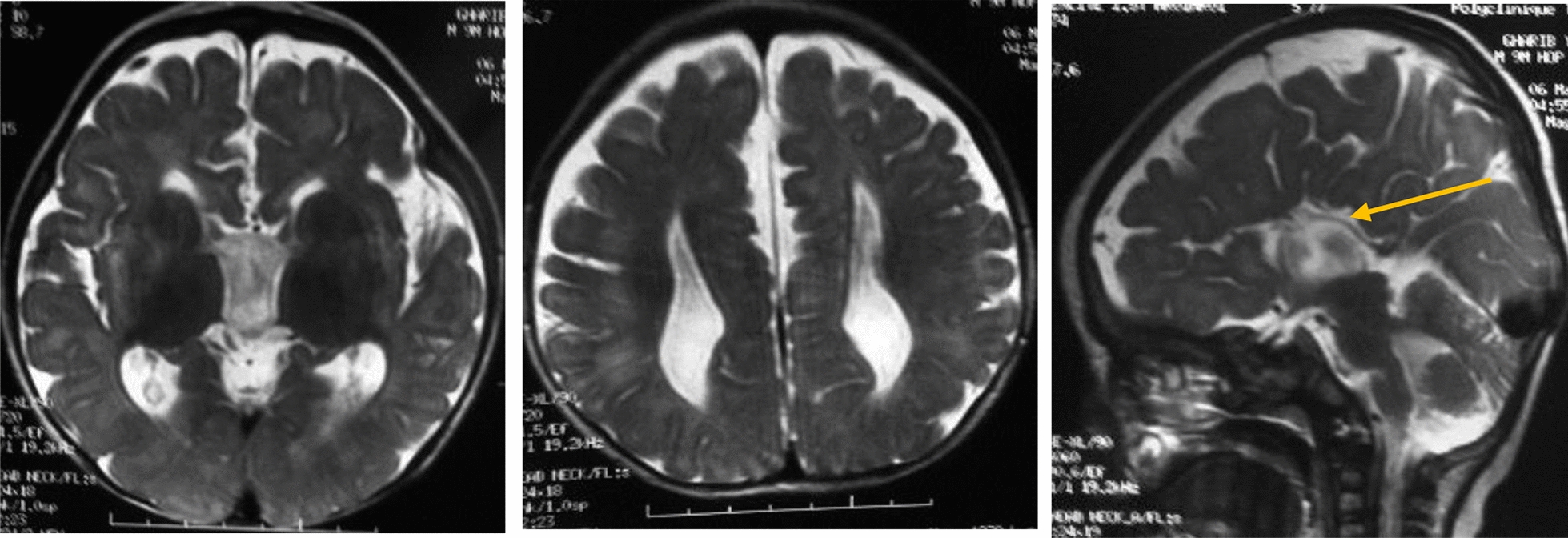
Patient 1 brain MRI showing*:* total callosal agenesis (yellow arrow) and brain atrophy associated with delayed encephalic myelination

Clinical examination showed microcephaly at − 3.3DS with obvious facial dysmorphia (Fig. 5): the forehead is short with protrusion of the metopic, and the palpebral slits are oblique downwards. There is a bilateral epicanthus with divergent strabismus on the left. The lips are thin with anomalies of the teeth: the canines are pointed with an absence of the middle incisors. The ears are high and badly hemmed. He also has a single palmar crease on the left. The left testicle was not palpated and an hypospadias was noted. An axial and peripheral hypotonia with a pyramidal syndrome was noted in the lower limbs as well as a ligamental hyperlaxity. Brain MRI (Fig. 6) showed a malformative encephalic anomaly with total callosal agenesis, quadriventricular ectasia, and cerebral atrophy predominantly frontotemporal without abnormalities of the cervical-occipital hinge and sellar region, associated with a delay in encephalic myelination. Cardiac ultrasound showed an interventricular septal defect associated with moderate pulmonary stenosis. The abdominal ultrasound returned normal.

#### Patient 2

At the first consultation, the infant was a 9-month-old boy from a non-consanguineous marriage with no family history. However, we discovered a pregnancy complicated by late intrauterine growth retardation (IUGR) discovered at the eighth month in his personal history. At the age of four months, the patient underwent surgery for an inguinal hernia and testicular ectopia. At 11 months, he shows a psychomotor delay in acquiring the sitting position. At the age of 2 years and 4 months, he presents convulsions and is under treatment of depakine. Microcephaly was detected at − 3.25 Ds with an obvious facial dysmorphia (Fig. 5): the forehead is short with protrusion of the metopic. There is a bilateral epicanthus with hypertelorism. The lips are thin with widely spaced teeth, and long lashes and the ears are high and badly hemmed. Polydactyly, micropenis, and axial hypotonia are also present in the infant. MRI showed hypoplasia of the corpus callosum and decreased volume of the periventricular white matter.

## Data Availability

Data and materials are available from the corresponding author and available upon request.

## Abbreviations

Array CGH: Array comparative genomic hybridization
*AKT3*: AKT serine/threonine kinase 3
CCA: Corpus callosum agenesis
CCM: Corpus callosum malformations
CC: Corpus callosum
*CEP170*: Centrosomal protein 170
DAPI: Diamino-2-phenylindole
DNA: Desoxyribonucleic acid
FISH: Fluorescence in situ hybridization
*FMN2*: Formin 2
HCC: Hypoplasia of the corpus callosum
*HNRNPU*: Heterogeneous nuclear ribonucleoprotein U
ISCN: International System for Human Cytogenetic Nomenclature 2020
OMIM: Online Mendelian Inheritance in Man
*PLD5*: Phospholipase D family member 5
*RGS7*: Regulator of G protein signaling 7
*SMYD3*: SET and MYND domain containing 3
*SDCCAG8*: SHH signaling and ciliogenesis regulator SDCCAG8
*ZNF238*: Zinc finger- and BTB domain-containing protein 18 (ZBTB18)
